# Childhood Maltreatment and BMI Trajectories to Mid-Adult Life: Follow-Up to Age 50y in a British Birth Cohort

**DOI:** 10.1371/journal.pone.0119985

**Published:** 2015-03-26

**Authors:** Chris Power, Snehal M. Pinto Pereira, Leah Li

**Affiliations:** Population, Policy and Practice, UCL Institute of Child Health, University College London, WC1N 1EH, London, United Kingdom; NIH / NIDDK, UNITED STATES

## Abstract

**Background:**

Childhood maltreatment including abuse and neglect has been associated with adult obesity, but evidence on life-course development of obesity or BMI gain is unclear. We aim to establish whether childhood maltreatments are related to obesity or BMI at different life-stages 7y-50y and to identify possible explanations for associations.

**Methods:**

Childhood physical, psychological and sexual abuse, neglect and BMI at seven ages were recorded in the 1958 birth cohort (n~15,000). Associations of child maltreatments with BMI at separate ages were tested using linear regression or logistic regression for obesity, and with rate of child-to-adult BMI gain using multilevel models. We adjusted for potential covariates.

**Results:**

Abuse was reported in ~12% of the population. Abuse was not associated with elevated childhood BMI, but adult associations were observed: i.e. the abused had faster child-adult BMI gain than the non-abused; associations were independent of adult covariates. For physical abuse in both genders there was a positive linear association of ~0.006/y zBMI gain with age after adjustment for all covariates. Similarly, there was a linear association of physical abuse with obesity risk: e.g. among females from a low OR_adjusted_ of 0.34 (0.16,0.71) at 7y to 1.67 (1.25,2.24) at 50y. In females faster zBMI gains with age of ~0.0034/y were observed for sexual abuse and increases in obesity risk were faster: from a low OR_adjusted_ of 0.23 (0.06,0.84) at 7y to 1.34 (0.86,2.10) at 50y. Psychological abuse and neglect associations were less consistent.

**Conclusions:**

Childhood maltreatment associations with BMI or obesity varied across life: physical and, in females, sexual abuse were associated with faster lifetime BMI gains, which may have detrimental long-term health consequences.

## Introduction

Childhood maltreatment including abuse and neglect has been linked to numerous health outcomes over the long-term such as mortality, chronic disease, obesity and poor mental health [1]. Whilst the impact of maltreatment on mental health is well-documented, there is more limited understanding of the association between childhood maltreatment and development of obesity over the life-course. The evolution of the association between maltreatment and obesity is of particular interest given that obesity is linked to mortality from cardiovascular disease[2] and thus, may be on the pathway from maltreatment to several long-term health outcomes, as suggested by research on chronic metabolic and immunity-related disease such as type 2 diabetes [3,4] and inflammation in mid-life [5].

Research on childhood maltreatment and obesity shows heterogenous results. However, a recent meta-analysis of 41 studies (190,285 participants) reported an elevated obesity risk in adulthood (OR adjusted for publication bias = 1.21 (1.12, 1.32)) but not in childhood in association with child maltreatment [6]. This finding highlights the need to consider Body Mass Index (BMI) at specific ages and changes in BMI throughout life. Evidence from the few studies available suggests that childhood maltreatment groups have greater BMI gains over phases of the life-span than others [4,7,8]. However, studies to date are limited to small selected populations focussing only on sexual abuse [7] or reliant on self-reported BMI measures [8] sometimes obtained retrospectively [4] and rarely extending beyond young adulthood [7,8].

Plausible mechanisms for a child maltreatment—adult obesity association have been identified. First, the well-established impact of child maltreatment on mental health [1] and co-morbidity of obesity and depressive symptoms [9] has led to the proposition that depression mediates in the association with obesity [6]. Second, given observations of child maltreatment with poor health behaviours, these in turn may lead to long-term health outcomes [10]. The latter possibility is complex for BMI/obesity where some behaviours such as smoking are associated with decreased rather than increased risk seen for disease outcomes [11]. Third, human and animal studies that show early-life stressors to be associated with altered brain responses suggest that there may be biologically embedded effects with the potential to influence long-term health risks [1, 12]. Research on child maltreatment and adiposity needs to take account of such complex relationships that evolve over the life-course. To our knowledge no study has examined child maltreatment in the general population and prospectively assessed BMI/obesity trajectories over long periods of life. Using data from a large nationwide cohort followed from birth to 50y, we aimed to establish whether: (i) child maltreatment (abuse and neglect) are associated with BMI or obesity at specific life-stages and with differing lifetime BMI or obesity trajectories child to mid-adulthood; (ii) associations are independent of other early-life or adult factors, e.g. disadvantaged material circumstances or health behaviours; and (iii) associations are independent of depressive symptom level.

## Methods

The 1958 cohort consists of all births during one week March 1958 in England, Scotland and Wales (n = 17,638) and a further 920 immigrants with the same birth week recruited to 16y [13]. Information was collected throughout child (7, 11,16y) and adulthood (23, 33, 45, 50y). At 45y, 11,971 individuals in contact with the study were invited to a home-based clinical assessment by a nurse; the 9,377 (78%) respondents were broadly representative of the total surviving cohort [14]. The 45y survey included questions about childhood maltreatment for which participants gave written informed consent.

### Ethics statement

Ethical approval was given by the South-East Multi-Centre Research Ethics Committee (MREC ref: 01/1/44).

### Childhood maltreatment

Neglect was measured from information collected in childhood (7y and 11y) during a home-based interview with the parent, usually the mother, and from a structured questionnaire completed by the child’s teacher. Scales (range 0–5) were derived by summing five items on the child’s physical appearance and parental involvement with the child at 7y and 11y (Table 1 footnotes). If one or two of five items were missing they were imputed (see below); if >2 items were missing, the score was missing. We created binary measures using a cut-off of ≥2 on separate 7y and 11y scales and for a summary variable for neglect at either 7 and/or 11y. Abuse by a parent in childhood to 16y was reported in adulthood (45y) using a confidential self-complete questionnaire with direct computer data entry. Questions were derived from the Personality and Total Health (PATH) Through Life Project [15], originating from the Parental Bonding Instrument [16], the British National Survey of Health and Development [17], and the US National Comorbidity Survey [18] (Table 1 footnotes). We created three binary variables: physical, psychological (emotional) and sexual abuse.

**Table 1:** Characteristics of the 1958 British birth cohort study.

	Males			Females		
Childhood maltreatment	N	(%)		N	(%)	
Abuse						
Physical	4621	5.95		4687	6.14	
Psychological	4622	8.29		4687	11.7	
Sexual	4621	0.48		4687	2.71	
All	4621	10.7		4687	13.9	
Neglect score						
≥2 at 7y and/or 11y*	7923	21.8		7501	18.5	
BMI (kg/m^2^)	N	mean (SD)	(IQR)^†^	N	mean (SD)	(IQR)^†^
7y	6873	15.9 (1.63)	(15.0,16.7)	6422	15.9 (1.91)	(14.6,16.7)
11y	6377	17.3 (2.41)	(15.8,18.2)	6114	17.6 (2.70)	(15.8,18.9)
16y	5563	20.3 (2.72)	(18.5,21.4)	5229	21.0 (2.96)	(19.0,22.5)
23y	6024	23.1 (2.90)	(21.2,24.5)	6057	22.1 (3.25)	(20.0,23.4)
33y	5408	25.6 (4.04)	(23.1,27.5)	5562	24.6 (4.97)	(21.4,26.5)
45y	4630	27.8 (4.37)	(25.0,30.1)	4677	27.0 (5.62)	(23.1,29.7)
50y	4180	28.1 (4.81)	(24.9,30.4)	4139	26.8 (5.60)	(22.9,29.5)
Obese^‡^ N (%)	N	(%)		N	(%)	
7y	6873	1.40		6422	2.44	
11y	6377	1.44		6114	1.64	
16y	5563	1.55		5229	1.49	
23y	6024	2.27		6057	3.07	
33y	5408	11.0		5562	12.0	
45y	4630	25.3		4677	23.7	
50y	4180	27.5		4139	23.0	

Table based on observed data, N varies due to missing data.

*Neglect at 7y and/or 11y (if one missing, other age used).

^†^ IQR = inter-quartile range

^‡^ defined as BMI≥20.63 at 7y, 25.10 at 11y, 28.88kg/m^2^ at 16y for males; ≥20.51, 25.42 and 29.4kg/m^2^ respectively in females; in adulthood BMI≥30kg/m^2^.

### BMI

Height and weight were measured using standard protocols (e.g. light indoor clothing with shoes removed) at 7y, 11y, 16y, 33y and 45y, and self-reported at 23y and 50y [19]. Childhood measures were obtained during a physical examination by trained medical personnel conducted at the child’s school, whilst all adult data were collected by trained interviewers (at 23y, 33y and 50y) or nurses (at 45y) in the participant’s home. BMI was calculated as weight/height^2^ (kg/m^2^) at each age. International BMI cut-offs were used to define obesity in childhood [20] and adulthood (details in Table 1).

### Covariates

Covariates were selected *a-priori* based on the literature. These included prospective parental reports (during interviews at ages indicated in Table footnotes) for parental characteristics (BMI), prenatal/infancy factors (birthweight, gestational age, maternal smoking during pregnancy, infant feeding) and childhood factors (socio-economic background, household crowding, amenities and tenure, ill-health, pubertal timing). During adulthood home-based interviews, participants reported their educational qualifications and at repeated ages 23–50y, their smoking, employment status, physical activity, alcohol consumption and depressive symptoms [21] (details in Table footnotes).

### Statistical analysis

In preliminary analyses we examined simple associations between childhood maltreatments and BMI at different ages in order to obtain a life-course overview before proceeding to longitudinal analyses of BMI trajectories. Specifically for our first aim, which was to assess associations between childhood maltreatments and BMI at different life-stages, we conducted unadjusted linear regression analysis separately for each maltreatment and BMI at each age 7y to 50y; likewise, using logistic regression we assessed associations with obesity at each age. Patterns of association were similar for neglect at 7y and 11y, hence we report results for the summary variable, neglect at 7y and/or 11y. A unit (kg/m^2^) of BMI has different implications in child and adulthood, so in subsequent analyses of BMI as a continuous variable, we internally standardised BMI to age and sex-specific SD scores to facilitate comparison across age.

In main analyses to establish whether child maltreatment groups had differing lifetime BMI trajectories from the non-maltreated, we performed longitudinal analyses of child to mid-adulthood BMI. Specifically, to examine associations between each maltreatment and zBMI trajectory we used multilevel (mixed-effects) models that allow for correlation between repeated zBMI measures on the same individuals over time. Models were fitted by assuming an unstructured variance-covariance matrix among repeated measures. Although the mean trajectory for zBMI for the cohort approximates a horizontal line centred at zero, models include a random intercept (zBMI at 7y, as age was centred at 7y) and random slope for zBMI (coefficient for linear age term, i.e. rate of change in zBMI/year), a maltreatment group and its interaction with age (as a fixed effect), to test whether the rate of change in zBMI varied by maltreatment. The quadratic term of age (i.e. age^2^) and its interaction with maltreatment measure were further added to the model to test whether the difference in average zBMI by maltreatment was a non-linear function of age. This latter term was significant and retained in models for neglect at 7/11y only. Thus, with age centred at 7y, the average rate of change over time is (coefficient for age + coefficient for neglect*age) +2*(coefficient for age^2^ + coefficient for neglect*age^2^) for those with neglect and (coefficient for age) +2*(coefficient for age^2^) for those without neglect.

We modelled associations with four levels of adjustment. First, we adjusted for exact age to allow for variations of several months at each follow-up. Second, we added adjustments for early-life factors. Third, we further adjusted for adult factors. Fourth, to assess whether any associations between child maltreatment and zBMI trajectory were independent of depressive symptom level, we further adjusted for time-varying depressive symptoms.

Maltreatment associations with obesity risk from 7y to 50y were examined using logistic regression with robust standard errors to allow for correlated within-individual obesity measures. We tested the interaction between maltreatment and age which (when exponentiated) represents the proportional change in the odds ratio (OR) for obesity/y of age. An interaction between maltreatment and age^2^ was examined; it was significant and retained in the model for psychological abuse among females only. We used the four levels of adjustment described above.

Sensitivity analyses were conducted. First, because childhood obesity was uncommon (<2.5%) we repeated all analyses using ≥95^th^ BMI or zBMI percentile at each age to test whether findings were influenced by obesity cut-offs. As expected, the 95^th^ centile cut-offs were lower than obesity cut-offs for ages 7 to 23y, but higher than obesity cut-offs for ages 33 to 50y (Table 1 and S1 Table). Second, because some maltreatments were correlated (i.e. for physical and psychological abuse r = ~0.5; in males, for sexual abuse with other abuse r = ~0.3) we conducted additional analyses adjusting for correlated maltreatments. Where relevant we report these findings.

From 17,638 participants enrolled at birth and immigrants by 7y (n = 378), 16,729 were alive and living in Britain at 7y; of these 16,639 participated (16,194 with data on neglect) at 7y or 11y. For childhood abuse, of 9,377 respondents at 45y, 9,315 provided relevant data. Multilevel models for participants with ≥1 BMI measure, 7y to 50y, were included: N = 15,424 for analysis of neglect at 7/11y; N~9310 for abuse. To minimize further data loss, missing covariates were imputed using MICE (multiple imputation chained equations) following current guidelines [22]. Imputation models included all model variables plus previously identified key predictors of missingness: i.e. cognitive ability and emotional status at 7y and class at birth [14]. Regression analyses were run across 10 imputed datasets. Imputed results were broadly similar to those using observed values; the former are presented. To facilitate comparison with our parallel studies of child maltreatment with height growth and pubertal timing[23,24] and with studies of BMI that show gender-specific results[7,8,25], all analyses were conducted for males and females separately.

## Results

Approximately 12% of participants reported childhood abuse, psychological abuse was the most and sexual abuse the least common; ~20% of participants had a childhood neglect score ≥2 at 7y and/or 11y (Table 1). Mean BMI increased throughout adulthood from 23.1 to 28.1kg/m^2^ in males and 22.1 to 26.8kg/m^2^ in females, ages 23 to 50y. Prevalence of obesity was <2.5% in childhood, but was much greater, at ~25% in mid-adulthood, 45 to 50y (Table 1).

**Fig 1:**
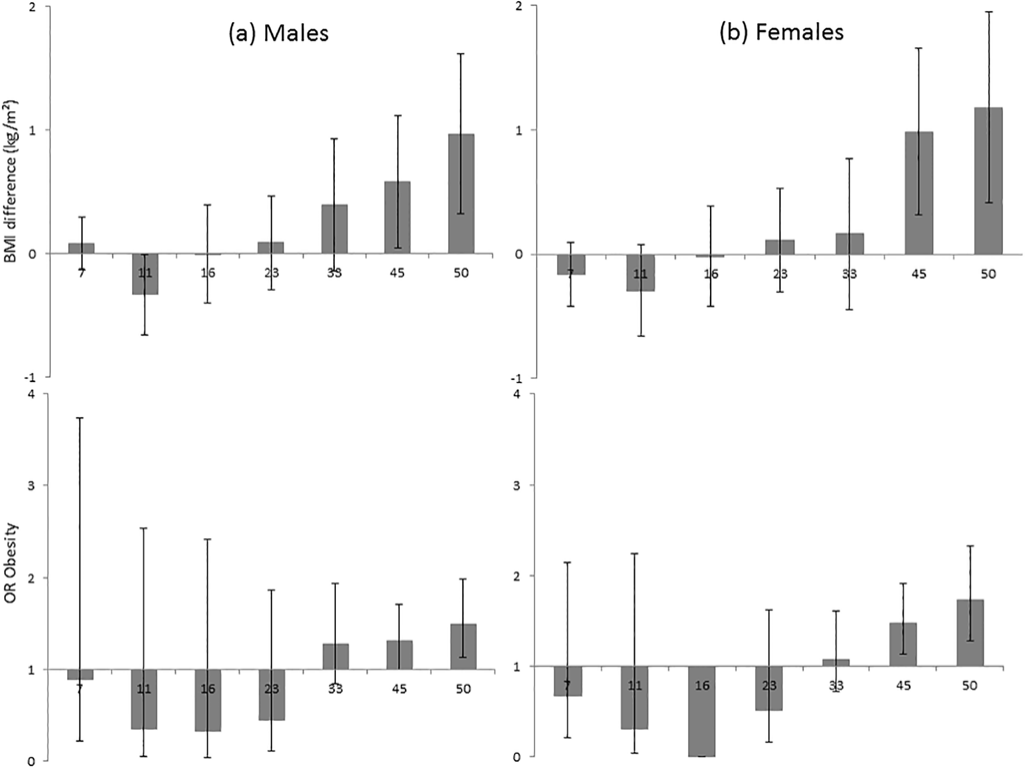
Difference in mean BMI (kg/m^2^) and OR for obesity (95% CIs) from 7 to 50y by physical abuse^†^ in males and females. Footnotes: ^†^ participant report in adulthood (45y) that they had been physically abused by a parent during their childhood before 16y, i.e. punched, kicked or hit or beaten with an object, or needed medical treatment.

Simple analyses of BMI at each age 7 to 50y showed a higher mean BMI in adulthood or OR for obesity in both sexes for neglect at 7/11y, physical and psychological abuse and, in females only, sexual abuse (Table 2). Pre-adolescent BMI, i.e. at 7 and 11y, was not elevated in association with abuse or neglect. Fig. 1 illustrates this pattern for physical abuse: e.g. for females, from no excess risk in childhood to an OR of 1.73 (1.28,2.32) at 50y. Thus for some maltreatments, simple analyses suggest a pattern from little difference to higher BMI with increasing age from child to mid-adulthood as seen in sensitivity analysis using ≥95^th^ BMI percentile for obesity (S1 Table).

**Table 2:** Mean difference (95%CI) in BMI (kg/m^2^) and OR (95% CI) for obesity at each age by childhood maltreatment (unadjusted).

	7y	11y	16y	23y	33y	45y	50y
	**Males**						
Abuse	**Mean difference (95%CI) in BMI (kg/m2)**						
Physical	0.08 (-0.13,0.30)	**-0.33 (-0.66,-0.01)**	0.00 (-0.40,0.40)	0.09 (-0.29,0.47)	0.40 (-0.14,0.93)	**0.58 (0.05,1.12)**	**0.97 (0.32,1.62)**
Psychological	0.17 (-0.01,0.35)	-0.12 (-0.40,0.16)	0.03 (-0.31,0.38)	0.09 (-0.24,0.42)	0.18 (-0.27,0.63)	0.23 (-0.23,0.69)	0.41 (-0.14,0.97)
Sexual	0.18 (-0.51,0.88)	1.15 (-0.03,2.33)	0.69 (-0.60,1.99)	0.93 (-0.47,2.32)	0.13 (-1.64,1.89)	-0.13 (-2.01,1.74)	0.10 (-2.19,2.39)
Neglect score							
≥2 at 7 and/or 11y	-0.08 (-0.18,0.02)	-0.11 (-0.26,0.03)	0.01 (-0.17,0.19)	**0.54 (0.35,0.73)**	**0.36 (0.09,0.63)**	**0.55 (0.20,0.90)**	**0.69 (0.29,1.08)**
Abuse	**OR (95%CI) obesity**						
Physical	0.90 (0.21,3.74)	0.35 (0.05,2.54)	0.33 (0.05,2.41)	0.45 (0.11,1.86)	1.28 (0.84,1.94)	**1.31 (1.00,1.71)**	**1.50 (1.13,1.99)**
Psychological	1.29 (0.46,3.66)	0.76 (0.23,2.45)	1.02 (0.37,2.86)	1.02 (0.44,2.38)	1.21 (0.84,1.74)	1.15 (0.91,1.45)	**1.36 (1.06,1.74)**
Sexual	0	5.44 (0.70,42.21)	4.11 (0.54,31.61)	3.54 (0.46,27.25)	1.04 (0.24,4.52)	1.48 (0.59,3.67)	1.44 (0.53,3.89)
Neglect score							
≥2 at 7 and/or 11y	0.64 (0.34,1.21)	1.17 (0.70,1.95)	**1.86 (1.16,2.98)**	**2.34 (1.61,3.40)**	1.17 (0.95,1.45)	**1.22 (1.02,1.45)**	**1.35 (1.14,1.61)**
	**Females**						
Abuse	**Mean difference (95%CI) in BMI (kg/m2)**						
Physical	-0.16(-0.42,0.09)	-0.29(-0.66,0.07)	-0.02(-0.42,0.38)	0.11(-0.31,0.53)	0.16(-0.44,0.77)	**0.99(0.31,1.66)**	**1.18(0.41,1.95)**
Psychological	-0.15(-0.34,0.04)	-0.17(-0.44,0.10)	-0.07(-0.37,0.24)	-0.01(-0.32,0.30)	-0.20(-0.65,0.25)	0.41(-0.09,0.92)	**0.61(0.06,1.16)**
Sexual	-0.14(-0.54,0.25)	-0.36(-0.93,0.20)	-0.27(-0.89,0.35)	0.27(-0.36,0.91)	-0.42(-1.35,0.52)	0.33(-0.67,1.32)	1.09(-0.06,2.24)
Neglect score							
≥2 at 7 and/or 11y	0.04 (-0.08,0.16)	0.03 (-0.15,0.21)	**0.29 (0.07,0.51)**	**0.65 (0.43,0.88)**	**0.78 (0.43,1.14)**	**1.15 (0.69,1.61)**	**1.40 (0.91,1.89)**
Abuse	**OR (95%CI) obesity**						
Physical	0.67 (0.21,2.15)	0.31 (0.04,2.24)	0^~^	0.51 (0.16,1.62)	1.07 (0.72,1.61)	**1.48 (1.14,1.91)**	**1.73 (1.28,2.32)**
Psychological	1.35 (0.71,2.58)	1.37 (0.64,2.92)	0.75 (0.27,2.09)	0.79 (0.40,1.58)	0.93 (0.67,1.27)	1.15 (0.94,1.42)	**1.44 (1.15,1.80)**
Sexual	0^~^	0^~^	0.88 (0.12,6.47)	0.83 (0.20,3.42)	0.92 (0.48,1.78)	1.15 (0.77,1.72)	**1.75 (1.13,2.71)**
Neglect score							
≥2 at 7 and/or 11y	0.99 (0.66,1.49)	1.41 (0.87,2.28)	**2.08 (1.21,3.55)**	**2.34 (1.68,3.27)**	**1.48 (1.21,1.81)**	**1.39 (1.16,1.66)**	**1.54 (1.28,1.86)**

^~^No females in these groups were obese.

As expected, childhood maltreatment was associated with several covariates known to be related to BMI (Table 3). Some covariates such as unemployment and smoking have been shown in the literature to be negatively associated with BMI, e.g. on average, smokers have a lower BMI [11]. In our study such characteristics were more common among maltreated groups: e.g., prevalence of smoking and unemployment 23y to 50y was higher in the maltreated than non-maltreated (Table 3). Potentially, these differences would lower the adult BMI among those exposed to childhood maltreatment. Allowance for such factors and their changes over time, as well as other covariates, is important for understanding associations with BMI at specific ages and BMI trajectories.

**Table 3:** Characteristics of those with no childhood maltreatment† and those abused or neglected (%).

		Abuse			Neglect score
	Non-maltreated^†^	Physical	Psychological	Sexual	≥2 at 7 and/or 11y
**Males**					
<O-level education	20.6	35.4**	28.7*	55.0*	58.8**
Unemployed at 23y	13.1	20.4*	18.8*	23.6	24.6**
Unemployed at 50y	8.38	18.2**	17.2**	39.6**	25.2**
Smoker at 23y	34.1	54.8**	49.0**	54.1	52.6**
Smoker at 50y	19.4	37.0**	31.1**	45.5*	38.0**
Physically inactive^#^ at 23y	56.2	59.5	59.3	51.8	61.4*
Physically inactive^#^ at 50y	27.6	33.7*	33.3*	42.7	37.8**
non-drinker at 23y	7.7	13.1*	12.7*	15.5	12.2**
≥22units/w at 23y	38.9	37.8	36.4	22.7	38.6
non-drinker at 50y	11.0	19.4**	19.0**	31.8*	27.5**
≥22units/w at 50y	40.0	39.8	37.0	22.7	38.8
**Females**					
<O-level education	21.1	34.5**	29.7**	45.1**	59.9**
Unemployed at 23y	28.3	49.4**	44.3**	53.2**	54.1**
Unemployed at 50y	15.4	24.9**	23.4**	26.9*	34.5**
Smoker at 23y	32.5	50.4**	48.3**	55.8*	52.9**
Smoker at 50y	19.7	37.0**	32.1**	46.8**	38.1**
Physically inactive^#^ at 23y	74.2	83.1*	79.2*	79.1	86.6**
Physically inactive^#^ at 50y	29.2	31.8	30.4	39.9*	38.1**
non-drinker at 23y	23.7	30.7*	29.4*	36.1*	39.2**
≥15units/w at 23y	13.1	12.2	11.8	12.1	8.75**
non-drinker at 50y	20.9	36.8**	29.0**	30.3*	40.2**
≥15units/w at 50y	18.9	21.3	17.9	21.0	17.9

Averaged across 10 imputed datasets †No neglect at 7y or 11y and no abuse

*p<0.05

** p<0.001 for each child maltreatment group vs non-maltreated

# <1/w

Modelled lifetime trajectories of zBMI and obesity risk (Table 4 and S2 Table) confirmed the patterns with age suggested by simple analyses.

### Childhood abuse

**Fig 2:**
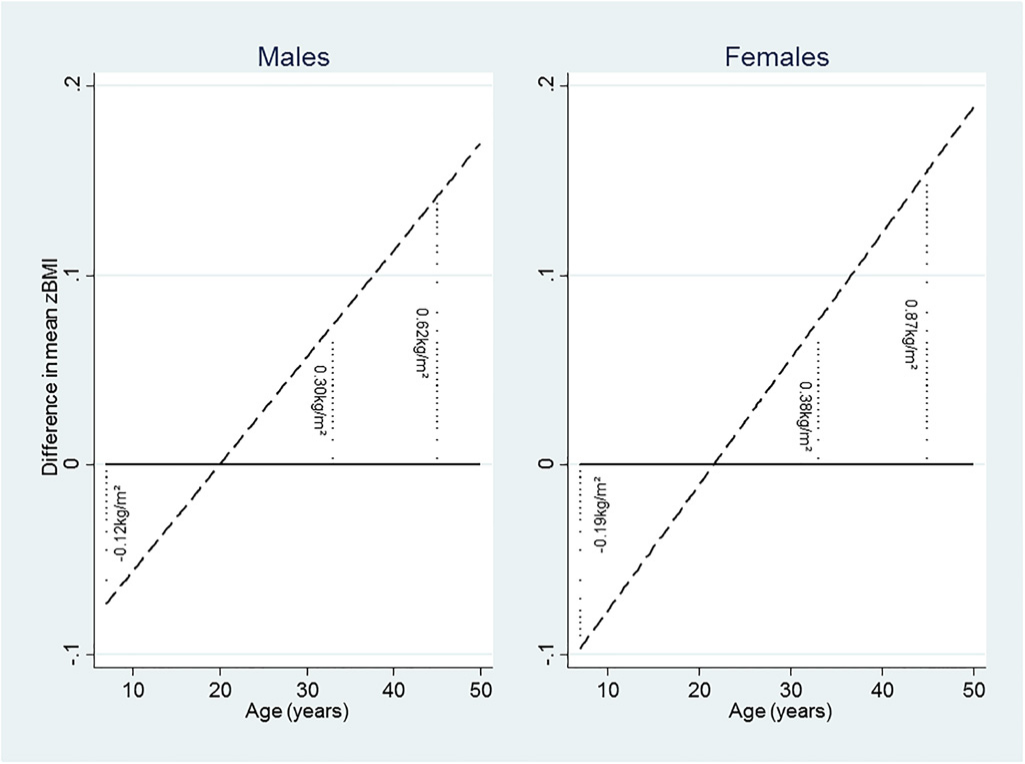
Difference in mean zBMI by childhood physical abuse^†^ from fully adjusted models, males and females*. Footnotes: ^†^ participant report in adulthood (45y) that they had been physically abused by a parent during their childhood before 16y, i.e. punched, kicked or hit or beaten with an object, or needed medical treatment. *Difference in mean zBMI by childhood physical abuse estimated from fully adjusted models; showing equivalent differences in BMI (kg/m^2^) at 7y, 33y and 45y. The positive linear association of zBMI gain with age and physical abuse is given as ~0.006/y (males) and ~0.007/y (females) in Table 4.

In both genders there was a positive linear association between zBMI gain with age and physical abuse, by ~0.006/y (males) and ~0.007/y (females) after adjustment for all covariates (Table 4). Fig. 2 illustrates this trend by showing the difference in zBMI with age, e.g. in males the difference was equivalent to 0.12kg/m^2^ lower at 7y, increasing with age to be greater by 0.62kg/m^2^ at 45y. There was also a faster increase in the OR_adjusted_ for obesity with age associated with physical abuse, in males by 1.03 (1.003,1.05) fold/y, from an OR_adjusted_ at 7y of 0.47, increasing to 0.71 at 23y, to 1.25 at 45y and 1.42 at 50y (S2 Table). For females, the OR for obesity associated with physical abuse increased by 1.04 (1.02,1.06) fold/y from an OR_adjusted_ at 7y of 0.34, to 0.61 at 23y, 1.39 at 45y to 1.67 at 50y. Simultaneous adjustment for psychological abuse had little effect on the zBMI or obesity estimates for physical abuse (S3 Table).

**Table 4:** Mean differences in zBMI (95% CIs) at 7y and rate of change in zBMI (7–50y) by childhood maltreatment, estimated using multilevel models.

Mean difference in 7y z-BMI or rate of zBMI change†	Unadjusted	Adjusted (A)*	Adjusted (A+B)**	Adjusted (A+B+C)***
**Males**				
**Physical abuse**				
7y z-BMI	-0.0503 (-0.1588,0.0583)	-0.0767 (-0.1805,0.0271)	-0.0737 (-0.1774,0.0300)	-0.0735 (-0.1772,0.0302)
rate of change in z-BMI	**0.0052 (0.0020,0.0085)**	**0.0052 (0.0020,0.0084)**	**0.0057 (0.0025,0.0089)**	**0.0057 (0.0024,0.0089)**
**Psychological abuse**				
7y z-BMI	0.0201 (-0.0728,0.1131)	0.0231 (-0.0660,0.1122)	0.0201 (-0.0684,0.1086)	0.0203 (-0.0681,0.1088)
rate of change in z-BMI	0.0011 (-0.0016,0.0039)	0.0011 (-0.0016,0.0039)	0.0015 (-0.0012,0.0043)	0.0014 (-0.0013,0.0042)
**Sexual abuse**				
7y z-BMI	0.2089 (-0.1611,0.5789)	0.0995 (-0.2554,0.4544)	0.0799 (-0.2742,0.4340)	0.0804 (-0.2736,0.4345)
rate of change in z-BMI	-0.0017 (-0.0128,0.0093)	-0.0016 (-0.0127,0.0094)	-0.0007 (-0.0118,0.0103)	-0.0009 (-0.0119,0.0101)
**Neglect 7 and/or 11**				
7y z-BMI	**-0.0883 (-0.1425,-0.0340)**	**-0.1488 (-0.2010,-0.0967)**	**-0.1612 (-0.2147,-0.1078)**	**-0.1605 (-0.2140,-0.1070)**
coefficient for interaction with age	**0.0156 (0.0109,0.0203)**	**0.0156 (0.0108,0.0204)**	**0.0167 (0.0120,0.0215)**	**0.0166 (0.0118,0.0213)**
coefficient for interaction with age^2^	**-0.0003 (-0.0004,-0.0002)**	**-0.0003 (-0.0004,-0.0002)**	**-0.0003 (-0.0004,-0.0002)**	**-0.0003 (-0.0004,-0.0002)**
**Females**				
**Physical abuse**				
7y z-BMI	-0.0876 (-0.1964,0.0212)	**-0.1132 (-0.2180,-0.0083)**	-0.0971 (-0.2005,0.0064)	-0.0969 (-0.2004,0.0066)
rate of change in z-BMI	**0.0066 (0.0034,0.0098)**	**0.0066 (0.0034,0.0098)**	**0.0068 (0.0036,0.0100)**	**0.0066 (0.0034,0.0098)**
**Psychological abuse**				
7y z-BMI	-0.0762 (-0.1576,0.0051)	**-0.0926 (-0.1711,-0.0142)**	-0.0593 (-0.1368,0.0182)	-0.0592 (-0.1368,0.0183)
rate of change in z-BMI	**0.0035 (0.0011,0.0059)**	**0.0035 (0.0011,0.0059)**	**0.0036 (0.0013,0.0060)**	**0.0035 (0.0011,0.0059)**
**Sexual abuse**				
7y z-BMI	-0.0601 (-0.2230,0.1027)	-0.0651 (-0.2238,0.0935)	-0.0790 (-0.2367,0.0787)	-0.0795 (-0.2371,0.0782)
rate of change in z-BMI	0.0034 (-0.0014,0.0082)	0.0033 (-0.0015,0.0081)	0.0036 (-0.0012,0.0084)	0.0034 (-0.0014,0.0082)
**Neglect 7 and/or 11**				
7y z-BMI	0.0039 (-0.0527,0.0605)	**-0.0728 (-0.1300,-0.0157)**	**-0.0634 (-0.1223,-0.0045)**	**-0.0622 (-0.1211,-0.0032)**
coefficient for interaction with age	**0.0131 (0.0087,0.0174)**	**0.0130 (0.0086,0.0173)**	**0.0129 (0.0086,0.0173)**	**0.0127 (0.0083,0.0170)**
coefficient for interaction with age^2^	**-0.0002 (-0.0003,-0.0001)**	**-0.0002 (-0.0003,-0.0001)**	**-0.0002 (-0.0003,-0.0001)**	**-0.0002 (-0.0003,-0.0001)**

†Mean difference in rate of change (i.e. additional rate of change associated with maltreatment) is represented by the coefficient for a linear age interaction term (and for 7y/11y neglect only it is a linear function of age: i.e. coefficient for interaction with age +2*(coefficient for interaction with age^2^)* age (where age is centred at 7y)

*A: adjusted for: social class at birth (or 7y if missing), identified from maternal reports, based on Registrar General’s classification of the father’s occupation: I&II (professional /managerial), IIINM (skilled non-manual), IIIM (skilled manual) and IV&V (semi-unskilled manual, including single-mother households), maternal smoking during pregnancy: smoking ≥1 cigarette/day after the 4th month of pregnancy recorded shortly after birth, mean parental zBMI: 1969 reported maternal and paternal BMI, standardised using internally derived standard deviation scores, mean parental z-BMI calculated as the average z-BMI of both parents (where missing, either mother or father zBMI was used), 7y amenities: having no access or sharing amenities (bathroom, indoor lavatory, and hot water supply), 7y household overcrowding: defined as ≥1.5 persons/room, 7y housing tenure: owner-occupied, council rented, private rental or other, birthweight: measured in ounces and converted into grams, gestational age (in weeks) estimated from the date of the mothers’ last menstrual period, breastfeeding reported in 1965 by the mother, categorized as ‘never’ or ‘ever’ breastfed, 7y ill health identified from medical examiner’s report of major handicap or disfiguring condition.

** A+B: adjusted as for A above + pubertal timing from parental report at 16y for age of voice change for males (three groups < = 12, 13–14, > = 15y) and menarche for females (five groups < = 11 to > = 15y), time-varying concurrent employment (in paid employed, others) 23–50y; educational qualifications by 50y (five groups: none, some, O-levels, A-levels or degree level); time-varying concurrent smoking 23–50y (non-smoker/ex-smoker/smoker); time-varying concurrent leisure-time physical activity frequency 23–50y (<1 vs ≥1 /week) which identifies those at elevated risk of all-cause mortality [44,45]; time-varying concurrent drinking 23–50y (males: non/infrequent drinker, 1–21, ≥22 units/week; females: non/infrequent drinker, 1–14, ≥15 units/week)

*** A+B+C: adjusted as above + time-varying depressive symptoms 23–50y (indicated by the 15 psychological items of the Malaise Inventory (8-items available at 50y were pro-rated to the 15 item scale used at other ages))

No association with zBMI or obesity trajectory was found for psychological abuse in males (Tables 4 and S2 Table) and an association among females disappeared with adjustment for physical abuse (S3 Table). In females but not males faster zBMI gains with age were observed for sexual abuse, by ~0.0034/y, although confidence intervals include 0. For obesity, sexual abuse was associated with a lower OR_adjusted_ at 7y of 0.23 (0.06,0.84) but faster, 1.04 (1.01,1.08) fold/y, linear increase with age such that the OR_adjusted_ increased to 0.44 at 23y, to 1.09 at 45y and 1.34 at 50y (S2 Table). This association attenuated slightly when adjusted for physical abuse (S3 Table).

### Childhood neglect

In both genders zBMI differences for neglected versus non-neglected groups varied with age. Neglect at 7y/11y was associated with a lower zBMI at 7y with estimated differences of 0.16 in males and 0.06 in females (equivalent to ~0.26 and 0.11kg/m^2^ respectively) and rate of zBMI gains varied non-linearly with age (Table 4). The difference in zBMI for neglect 7/11y changed from deficit at 7y to excess by 16y in females and 23y in males; the excess was maximal at 33y, with a 0.09 (0.03,0.14) and 0.12 (0.07,0.18) higher zBMI respectively in males and females. Similar differences with age were found for obesity using ≥95^th^ BMI percentile (data not presented). However, there were no corresponding changes with age for neglect for risk of obesity (S2 Table) and additional analyses for separate ages showed that for all except 23y, elevated obesity risks disappeared when adjusted for covariates: e.g. among females, an OR for obesity at 45y of 1.39(1.16,1.66) reduced to 1.06(0.86,1.30).

## Discussion

There are three major findings of our study. First, childhood maltreatment associations with BMI varied by age, highlighting the importance of considering BMI changes over the life-course. For some maltreatments notably physical abuse and neglect, and in females sexual abuse, BMI in childhood was lower or no different from the non-maltreated, but BMI became elevated by mid-adulthood following a faster rate of gain over the intervening period. In some instances, changes in BMI were marked: e.g., in physically abused females the OR_adjusted_ for obesity reversed from 0.34 at 7y to 1.67 at 50y. Second, not all childhood maltreatments showed consistent associations with BMI or obesity (e.g. psychological abuse). Third, we found differences in BMI-related socio-demographic and lifestyle factors for maltreatment groups compared to others, yet adjustment for several adult covariates had little effect on child maltreatment—BMI or obesity associations.

Study strengths include nationwide coverage and long follow-up. To our knowledge, no previous study has examined BMI trajectories for childhood abuse and neglect in a general population over more than four decades of life. All BMI measures were obtained prospectively, avoiding problems associated with recall. Most were based on measurements rather than self-report and it is unlikely that the latter could account for differing BMI trajectories because the differences were evident with measured BMIs in child and adulthood. Obesity prevalence was low in childhood, but findings were mostly supported by sensitivity analysis with a 95^th^ percentile cut-off and by analysis of BMI as a continuous variable. Extensive early life and contemporary covariates were measured prospectively, including some such as pubertal timing that have been overlooked in previous research. We took account of different covariates at several time-points to allow for changes in lifestyles and mental health that could affect variations of BMI with age. For childhood maltreatment, neglect was recorded prospectively at 7 and 11y based on multiple sources (parent and teacher report) that may reduce misclassification [26]. Rather than rely on individual items, which may not imply neglectful behaviour, we used a score of at least two items. Our neglect indicators correspond to conventional definitions (e.g. failure to meet a child’s basic physical, emotional, medical, or education needs)[27], although aspects such as failure to provide adequate nutrition or shelter are not covered. Information was not available on abuse by individuals other than a parent and on abuse after age 16y and given that childhood abuse was ascertained from adult reports we could not determine temporal order of abuse and BMI in childhood/adolescence. Study power to detect associations with sexual abuse was limited especially in males. All ascertainment methods for child maltreatment have limitations [27]. For example, parental report may be influenced by socially desirable responding and concealment, whilst adult report is subject to recall bias [28]. Retrospective report is an accepted method in population studies [27] and was blind to knowledge of issues to be investigated. Reassuringly, our previous work shows expected associations indicating construct validity, e.g. gender patterns, co-occurrence and links with family circumstances,[29] and mental health outcome [30]. For correlated maltreatments, we used simultaneously adjusted models. Despite control for lifetime covariates, unmeasured factors could partly account for associations. Also, we cannot discount the possibility that adjustment for potential mediators such as smoking and physical activity may induce biases, for example if there is exposure-mediator interaction[31]. As with any long-term study, sample attrition had occurred. To maximise available data, multilevel models included those with ≥1 BMI measure and we used multiple imputation to avoid loss due to missing data on covariates. Imputation assumes data are missing at random, this assumption is made reasonable because we included in imputation models previously identified key predictors of missingness [14].

Our study advances the literature in several ways. First, findings that associations of child maltreatment with BMI vary by age may explain some of the heterogeneity in the literature. In our population, child abuse or neglect was not associated with excess childhood BMI, but higher BMIs were observed in adulthood in line with estimates (OR = 1.21 to 1.36) from a recent overview [6]. From this overview it was possible to discern contrasting associations for child and adult BMI and implicit faster BMI gain because of the differing ages of included studies [6]. We confirm child and adult BMI patterns and track BMI gain within a single population. Faster BMI gain over several decades of life seen for child maltreatment agrees with the few existing studies over shorter age intervals [7,8]. Our findings suggest that the obesity focus in much of the literature may be insufficient to detect the faster BMI gains for child maltreatments which can occur in the absence of obesity.

Second, our results provide insights for the direction of child maltreatment-excess adult BMI associations. Establishing direction of association is complex, yet studies that examine several ages from early life can indicate whether excess BMI preceded maltreatment exposure. If heavier children were more likely to be abused or neglected, an association in adulthood may arise from a reverse direction of association; e.g. heavier females may be more likely to be sexually abused and thence remain heavier in adulthood. In line with others, [25] our findings did not support a reverse direction of association. Moreover, in parallel studies we found little evidence for child maltreatment associations with advanced maturation or height growth, which would be expected if maltreatment groups had been heavier in childhood [23,24]. Few if any studies examine pubertal timing in relation to child maltreatment and BMI; we found associations to be little altered with adjustment for this factor, even for neglect wherein differences in BMI gain appeared to coincide with puberty.

Third, our results shed light on the role of depression in childhood maltreatment—BMI associations. Child maltreatment is known to increase the risk of adult depression [1] and some reports suggest that depression is associated with elevated BMI [9] although the causal direction is unclear. If the direction is from obesity to depression rather than the reverse, as suggested by previous work on this cohort [9], then adjustment of child maltreatment—obesity associations may be inappropriate. Moreover, if depression contributes to child maltreatment—obesity associations we would expect stronger associations for psychological than physical abuse because of its stronger association with later depression [1]. Contrary to this expectation, findings here and elsewhere [6] suggest that psychological abuse or emotional neglect have weak inconsistent associations with BMI. In addition, our study found negligible effects on child maltreatment—BMI (or obesity) associations of adjustment for concurrent depressive symptoms at four adult ages. This finding suggests that child maltreatment-BMI (or obesity) associations are independent of depressive symptoms. However, it is possible that other psychological processes or poorer health behaviour could contribute to the association. Our finding that smoking rates were higher for most child maltreatment groups is consistent with recent meta-analyses, although evidence for alcohol use is less consistent [1]. Yet, in our study associations with BMI and obesity remained for some child maltreatments after allowing for smoking, physical activity and alcohol consumption. More evidence is needed from life-course studies to confirm our findings on the contribution of psychological and lifestyle factors to child maltreatment—BMI (or obesity) associations.

Further insights may emerge from a greater focus on physical abuse because of its consistent associations in both genders, with faster BMI or obesity trajectory and elevated adult BMI, and on sexual abuse for females. It is possible that their lower childhood BMI may reflect a greater vulnerability of lighter children to assault and/or under-nutrition, although there is little evidence for delayed growth or maturation of these groups [23,24]. The rapid BMI gain in adulthood for physical (and female sexual) abuse more than compensates for childhood BMI deficits, even after allowing for characteristics such as socio-economic background associated with rapid BMI gain in this population [19]. Epigenetic mechanisms may underpin child maltreatment–BMI links [32,33] or mark occurrence of early maltreatment [34]. In a sub-sample of this cohort, methylation differences were found for ‘any abuse’, including hypermethylation of *PM20D1* [35] reported previously to be associated with obesity [36]. Pending further understanding of mechanisms, our study adds to the literature on BMI trends with age. Our results agree with the Nurses’ Health Study II for severe physical and sexual abuse (8.7% and 5.5% of women) showing increasing excess BMI with age compared to non-abused [4], although a study of highly prevalent (~30%) physical abuse found no trend [8]. Elsewhere, child physical but not sexual abuse predicted higher adult BMI [37], but many studies of sexual abuse including ours, are hampered by low prevalence; even so, age trends and gender differences suggested by our results are similar to others [8, 25]. Our findings for childhood neglect agree with a US study showing faster BMI gain, 15 to 28y [8] and a Danish study showing higher obesity risk in young adulthood (~20y) using similar parental care measures to ours [38]; whereas for court-substantiated neglect in the US, no excess BMI was seen at 31y [37]. Whilst differences in neglect measures may account for some discrepancies, our study suggests that associations vary with age, although reasons for this variation with age are unknown.

Childhood maltreatment groups differed from their contemporaries in many aspects of their lives, such as lower qualifications and higher unemployment /smoking rates, 23y to 50y. In parallel, some maltreatment groups had lower BMI in childhood, followed by a faster rate of BMI gain and higher adult BMI. Because associations for child and adult BMI can be in opposite directions, studies of specific ages may not capture the full association of maltreatment with BMI and obesity. Child maltreatment has been linked to multiple long-term outcomes including several chronic diseases [1]. One plausible pathway through which adult health may be affected is via obesity, [3–5] and excess BMI gain. BMI gain is important because even within the normal BMI range it has been linked to adverse health outcomes [39–43]. Hence, the faster BMI trajectory for some child maltreatments may have detrimental health consequences in the long-term. Not all child maltreatments showed consistent associations with BMI or obesity (e.g. psychological abuse) hence, summary maltreatment measures may be inadequate to investigate long-term relationships with BMI or obesity. This is a study of one cohort and results may differ in other populations given their prevalence of child maltreatment or obesity. Future studies are needed to track long-term outcomes of child maltreatment, identify factors that may remedy adverse outcomes, monitor younger generations and support efforts aimed at primary prevention.

## Supporting Information

S1 Table OR (95% CI) for obesity (≥95th percentile) at each age by childhood maltreatment (unadjusted). (DOCX)

S2 Table Changing Odds ratio (OR) (95%CIs) for obesity with age for childhood maltreatments. (DOCX)

S3 Table (1) Mean differences in zBMI (95% CIs) at 7y and rate of change in zBMI (7–50y) and (2) Changing Odds ratio (OR) (95%CIs) for obesity with age in Females. (DOCX)
