# Supplementary material for: Childhood Maltreatment and BMI Trajectories to Mid-Adult Life: Follow-Up to Age 50y in a British Birth Cohort
Source: PLoS One. 2015 Mar 26;10(3):e0119985. doi: 10.1371/journal.pone.0119985 (PMC4374764; doi:10.1371/journal.pone.0119985)
Supplement: S1 Table — (DOCX) [file pone.0119985.s001.docx]

**Supplementary Table 1: OR (95% CI) for obesity (≥95th percentile) at each age by childhood maltreatment (unadjusted)**

|  | 7y | 11y | 16y | 23y | 33y | 45y | 50y |
| --- | --- | --- | --- | --- | --- | --- | --- |
|  | **Males** | | | | | | |
| *95^th^ percentile (kg/m²)* | *≥18.75* | *≥21.99* | *≥25.11* | *≥28.26* | *≥32.49* | *≥35.64* | *≥36.32* |
| Abuse |  |  |  |  |  |  |  |
| Physical | 0.95 (0.48,1.88) | 0.19 (0.05,0.78) | 0.88 (0.42,1.81) | 0.71 (0.33,1.53) | 0.90 (0.45,1.79) | 1.28 (0.77,2.12) | 1.39 (0.80,2.39) |
| Psychological | 0.96 (0.54,1.71) | 0.88 (0.48,1.60) | 1.16 (0.66,2.04) | 0.81 (0.43,1.51) | 0.82 (0.45,1.50) | 0.94 (0.57,1.54) | 1.03 (0.61,1.74) |
| Sexual | 1.20 (0.16,9.04) | 1.49 (0.19,11.39) | 1.25 (0.16,9.49) | 1.51 (0.20,11.58) | 0 | 2.00 (0.46,8.63) | 1.18 (0.16,8.97) |
| Neglect score |  |  |  |  |  |  |  |
| ≥2 at 7 and/or 11y | 0.74 (0.53,1.02) | 0.95 (0.72,1.25) | 1.09 (0.81,1.47) | 1.92 (1.49,2.47) | 1.25 (0.92,1.70) | 1.31 (0.93,1.83) | 1.52 (1.09,2.14) |
|  | **Females** | | | | | | |
| *95^th^ percentile (kg/m²)* | *≥ 19.31* | *≥ 22.94,* | *≥ 26.38,* | *≥ 28.17* | *≥ 34.53* | *≥ 37.96,* | *≥ 37.50* |
| Abuse |  |  |  |  |  |  |  |
| Physical | 0.38 (0.14,1.03) | 0.48 (0.20,1.19) | 0.50 (0.20,1.22) | 1.27 (0.70,2.32) | 1.36 (0.78,2.39) | 1.55 (0.97,2.46) | 1.61 (0.94,2.73) |
| Psychological | 0.86 (0.52,1.43) | 0.80 (0.47,1.35) | 0.83 (0.48,1.43) | 1.19 (0.75,1.88) | 0.87 (0.53,1.44) | 1.31 (0.90,1.90) | 1.24 (0.81,1.90) |
| Sexual | 0.23 (0.03,1.69) | 0.73 (0.23,2.35) | 0.77 (0.24,2.47) | 1.79 (0.82,3.93) | 0.44 (0.11,1.81) | 1.12 (0.51,2.42) | 2.12 (1.05,4.28) |
| Neglect score |  |  |  |  |  |  |  |
| ≥2 at 7 and/or 11y | 1.00 (0.74,1.34) | 0.95 (0.69,1.31) | 1.67 (1.22,2.30) | 2.23 (1.71,2.92) | 1.59 (1.19,2.13) | 1.52 (1.09,2.13) | 1.65 (1.17,2.33) |
