# Supplementary material for: Childhood Maltreatment and BMI Trajectories to Mid-Adult Life: Follow-Up to Age 50y in a British Birth Cohort
Source: PLoS One. 2015 Mar 26;10(3):e0119985. doi: 10.1371/journal.pone.0119985 (PMC4374764; doi:10.1371/journal.pone.0119985)
Supplement: S2 Table — (DOCX) [file pone.0119985.s002.docx]

**Supplementary Table 2: Changing Odds ratio (OR) (95%CIs) for obesity with age for childhood maltreatments**

| **Males** | Unadjusted | Adjusted (A)* | Adjusted (A+B)** | Adjusted (A+B+C)*** |
| --- | --- | --- | --- | --- |
| **Physical abuse** |  | | | |
| OR for obesity at 7y | 0.56 (0.24,1.30) | 0.48 (0.20,1.13) | 0.47 (0.20,1.12) | 0.47 (0.20,1.12) |
| Ratio of OR per year~ | 1.02 (1.00,1.04) | 1.03 (1.00,1.05) | 1.03 (1.00,1.05) | 1.03 (1.00,1.05) |
| **Psychological abuse** |  | | | |
| OR for obesity at 7y | 0.98 (0.49,1.97) | 0.93 (0.46,1.90) | 0.95 (0.47,1.93) | 0.95 (0.47,1.93) |
| Ratio of OR per year~ | 1.01 (0.99,1.02) | 1.01 (0.99,1.03) | 1.01 (0.99,1.03) | 1.01 (0.99,1.03) |
| **Sexual abuse** |  | | | |
| OR for obesity at 7y | 3.06 (0.27,34.19) | 2.08 (0.17,25.26) | 2.12 (0.18,25.32) | 2.12 (0.18,25.48) |
| Ratio of OR per year~ | 0.98 (0.92,1.04) | 0.98 (0.92,1.05) | 0.98 (0.92,1.05) | 0.98 (0.92,1.05) |
| **Neglect 7 and/or 11** |  | | | |
| OR for obesity at 7y | 1.24 (0.89,1.74) | 0.98 (0.69,1.38) | 0.94 (0.67,1.33) | 0.94 (0.67,1.33) |
| Ratio of OR per year~ | 1.00 (0.99,1.01) | 1.00 (0.99,1.01) | 1.00 (0.99,1.01) | 1.00 (0.99,1.01) |
| **Females** |  |  |  |  |
| **Physical abuse** |  |  |  |  |
| OR for obesity at 7y | 0.39 (0.19,0.79) | 0.34 (0.16,0.69) | 0.34 (0.16,0.72) | 0.34 (0.16,0.71) |
| Ratio of OR per year~ | 1.04 (1.02,1.05) | 1.04 (1.02,1.06) | 1.04 (1.02,1.06) | 1.04 (1.02,1.06) |
| **Psychological abuse**^#^ |  |  |  |  |
| OR for obesity at 7y | 1.52 (0.59,3.90) | 1.45 (0.56,3.73) | 1.60 (0.61,4.22) | 1.61 (0.61,4.25) |
| exp(coefficient for interaction with age) | 0.95 (0.90,1.02) | 0.95 (0.89,1.02) | 0.95 (0.88,1.01) | 0.95 (0.89,1.01) |
| exp(coefficient for interaction with age^2^) | 1.00 (1.00,1.002) | 1.00 (1.00,1.002) | 1.00 (1.00,1.002) | 1.00 (1.00,1.002) |
| **Sexual abuse** |  |  |  |  |
| OR for obesity at 7y | 0.30 (0.08,1.05) | 0.26 (0.07,0.93) | 0.23 (0.06,0.85) | 0.23 (0.06,0.84) |
| Ratio of OR per year~ | 1.04 (1.01,1.07) | 1.04 (1.01,1.07) | 1.04 (1.01,1.08) | 1.04 (1.01,1.08) |
| **Neglect 7 and/or 11** |  |  |  |  |
| OR for obesity at 7y | 1.48 (1.09,2.02) | 1.13 (0.82,1.55) | 1.09 (0.79,1.51) | 1.09 (0.79,1.51) |
| Ratio of OR per year~ | 1.0003 (0.992,1.009) | 1.001 (0.992,1.010) | 1.001 (0.992,1.01) | 1.001 (0.992,1.01) |

~ Ratio of the OR for obesity at age x+1 vs OR for obesity at age x. For example: for physical abuse in males, the OR_adjusted_ for obesity at 7y was 0.47, increasing to 0.48 (i.e 0.47
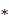

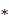
1.026) at 8y, and to 0.47
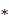

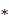
1.026^(x-7)^ at age x

^#^Non-linear association: coefficients for interaction with age and age^2^. For example, for psychological abuse in females, the OR_adjusted_ for obesity at 7y was 1.61, changing to 1.53 (i.e. 1.61
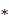

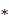
 (0.9472
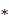

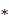
1.0012) at age 8y and to
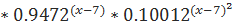
 1.61
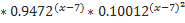

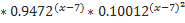
at age x.

*A: adjusted for: social class at birth, birthweight, mean parental zBMI, 7y amenities, 7y household overcrowding, 7y housing tenure, gestational age, breastfeeding, 7y ill health, maternal smoking

** A+B: adjusted as for A above + puberty (age voice breaks males, age at menarche females), concurrent employment 23-50y; 50y educational attainment; concurrent smoking 23-50y; concurrent physical activity 23-50y; concurrent drinking 23-50y

*** A+B+C: adjusted as above + concurrent malaise: 23-50y; 50y 8-items prorated to the 15 item scale used at other ages
