# Supplementary material for: Childhood Maltreatment and BMI Trajectories to Mid-Adult Life: Follow-Up to Age 50y in a British Birth Cohort
Source: PLoS One. 2015 Mar 26;10(3):e0119985. doi: 10.1371/journal.pone.0119985 (PMC4374764; doi:10.1371/journal.pone.0119985)
Supplement: S3 Table — (DOCX) [file pone.0119985.s003.docx]

**Supplementary Table 3: (1) Mean differences in zBMI (95% CIs) at 7y and rate of change in zBMI (7-50y) and (2) Changing Odds ratio (OR) (95%CIs) for obesity with age in Females.**

Models are adjusted for covariates (A in Tables 4 and S2)^†^ and additionally adjusted for other specified abuse.

|  | (1) Mean differences in zBMI (95% CIs) at 7y and rate of change in zBMI | (2) Changing Odds ratio (OR) (95%CIs) for obesity with age |
| --- | --- | --- |
| **Physical abuse** |  |  |
| 7yr z-BMI or OR for obesity at 7y | **-0.1132 (-0.2180,-0.0083)** | **0.3354 (0.1636,0.6880)** |
| Rate of change in z-BMI or Ratio of OR per year~ | **0.0066 (0.0034,0.0098)** | **1.0370 (1.0176,1.0566)** |
| ***Additionally adjusted for psychological abuse*** |  |  |
| 7yr z-BMI or OR for obesity at 7y | -0.0656 (-0.1890,0.0577) | **0.3148 (0.1370,0.7234)** |
| Rate of change in z-BMI or Ratio of OR per year~ | **0.0057 (0.0019,0.0095)** | **1.0383 (1.0166,1.0605)** |
| **Psychological abuse**^#^ |  |  |
| 7yr z-BMI | **-0.0926 (-0.1711,-0.0142)** |  |
| Rate of change in z-BMI | **0.0035 (0.0011,0.0059)** |  |
| ***Additionally adjusted for physical abuse*** |  |  |
| 7yr z-BMI | -0.0669 (-0.1592,0.0255) |  |
| Rate of change in z-BMI | 0.0012 (-0.0016,0.0041) |  |
|  |  |  |
| OR for obesity at 7y |  | 1.4465 (0.5608,3.7309) |
| exp(coefficient for interaction with age) |  | 0.9521 (0.8928,1.0152) |
| exp(coefficient for interaction with age^2^) |  | **1.0011 (1.0000,1.0021)** |
| ***Additionally adjusted for physical abuse*** |  |  |
| OR for obesity at 7y |  | 2.0477 (0.7685,5.4566) |
| exp(coefficient for interaction with age) |  | 0.9440 (0.8859,1.0058) |
| exp(coefficient for interaction with age^2^) |  | 1.0010 (0.9999,1.0020) |
| **Sexual abuse** |  |  |
| 7yr z-BMI or OR for obesity at 7y | **0.2609 (0.0731,0.9314)** | **0.2609 (0.0731,0.9314)** |
| Rate of change in z-BMI or Ratio of OR per year~ | **1.0396 (1.0073,1.0730)** | **1.0396 (1.0073,1.0730)** |
| ***Additionally adjusted for physical abuse*** |  |  |
| 7yr z-BMI or OR for obesity at 7y | 0.3663 (0.0988,1.3583) | 0.3663 (0.0988,1.3583) |
| Rate of change in z-BMI or Ratio of OR per year~ | 1.0273 (0.9947,1.0610) | 1.0273 (0.9947,1.0610) |

^†^ estimated using multilevel models and including adjustments (model A in Tables 4 and S2) for: social class at birth, birthweight, mean parental zBMI, 7y amenities, 7y household overcrowding, 7y housing tenure, gestational age, breastfeeding, 7y ill health, maternal smoking

~ Ratio of the OR for obesity at age x+1 vs OR for obesity at age x. (see note for Table S2 for an example).

^#^Non-linear association for OR (see note for Table S2)
